# Gamma-Ray Spectral Unfolding of CdZnTe-Based Detectors Using a Genetic Algorithm

**DOI:** 10.3390/s20247316

**Published:** 2020-12-19

**Authors:** Nicola Sarzi Amadè, Manuele Bettelli, Nicola Zambelli, Silvia Zanettini, Giacomo Benassi, Andrea Zappettini

**Affiliations:** 1 IMEM/CNR, Parco Area Delle Scienze 37/A, 43100 Parma, Italy; manuele.bettelli@imem.cnr.it (M.B.); andrea.zappettini@imem.cnr.it (A.Z.); 2 due2lab s.r.l., Via Paolo Borsellino 2, Scandiano, 42019 Reggio Emilia, Italy; zambelli@due2lab.com (N.Z.); zanettini@due2lab.com (S.Z.); benassi@due2lab.com (G.B.)

**Keywords:** gamma spectrum, unfolding algorithm, genetic algorithm

## Abstract

The analysis of γ-ray spectra can be an arduous task, especially in the case of room temperature semiconductor detectors, where several distortions and instrumental artifacts conceal the true spectral shape. We developed a genetic algorithm to perform the unfolding of γ-spectra in order to restore the true energy distribution of the incoming radiation. We successfully validated our approach on experimental spectra of four radionuclides (${}^{241}$Am, ${}^{57}$Co, ${}^{137}$Cs and ${}^{133}$Ba) acquired with two CdZnTe-based detectors with different contact geometries (single pixel and drift strip). The unfolded spectra consist of δ-like peaks in correspondence with the radiation emissions of each radioisotope.

## 1. Introduction

For many years, experimental scientists have pursued the goal of extracting the ground truth from a measurement by removing the instrumental response function. This attempt is fundamental in every field where the relevant information may be concealed or distorted by non-ideal behavior of the measuring instrument, such as the one of room temperature semiconductor detectors (RTSD). RTSDs are largely employed today for medical and astrophysical applications [1], as well as for environmental monitoring [2] and for high flux applications [3,4]. The main source of distortion is related to those events of radiation interaction for which the total energy deposited is inferior to the real energy of the incoming photon. These events produce several specific features in the final spectrum: fluorescence and escape peaks, Compton edges, backscattered peaks, and pair production peaks. Such additional features clearly appear in the spectrum of a mono-energetic incident radiation, but they are hard to distinguish in the case of a multi-energy radioactive source or a continuous incident spectrum. Secondly, the photogenerated charges may undergo an incomplete collection due to carrier trapping, which leads to asymmetric peaks (“hole tailing”); such incomplete collection depends on defect concentration, contacts geometry, and crystal thickness. Finally, RTSDs are characterized by a poorer signal-to-noise ratio than cooled Si- or Ge-based detectors, which leads to broad and overlapped lines. Since most common isotope identification algorithms are based on peak search methods, all of these features represent an obstacle to a straightforward data analysis. Although advanced technological solutions can be adopted to improve the overall performances of these devices [5,6], in some applications, compactness and cost-effectiveness of the whole system are of primary concern, leading to limited spectral performances.

The so-called spectral unfolding or spectral deconvolution consists of removing all the distortions mentioned above from a measured spectrum and restoring the real energy distribution of the incident radiation [7]. In the case of γ-spectra, this process is able to extract δ-like peaks which correspond to the characteristic emissions of a given radionuclide. In this way, spectral unfolding drastically improves the output spectra of devices with low or average energy resolution, where different peaks often overlap owing to poor signal-to-noise ratio. As a matter of fact, the final goal of analyzing γ-spectra is to identify the radionuclides which produced it: this is a typical pattern recognition problem that can be addressed with various approaches (e.g., peak search and match, template matching, regions of interest) [8]. Spectral unfolding could ease this task by removing undesired features and bringing out the relevant ones. This can be achieved by solving the Fredholm integral equation of the first kind which describes the response of the instrument:

(1) $$S^{\prime }\left(E^{\prime }\right)=\int_{0}^{\infty }R\left(E^{\prime },E\right)S\left(E\right)dE$$

where $S^{\prime }\left(E^{\prime }\right)$ is the measured energy distribution, S(E) is the real energy distribution, and *R* is the response function of the detector. The meaning of $R\left(E^{\prime },E\right)$ is straightforward: it represents the probability density function (PDF) that an incoming photon of energy *E* is measured with an energy $E^{\prime }$. For actual measurements, spectra are discretized, that is, $S^{\prime }$ and *S* are vectors, and *R* is a matrix:

(2) $$\vec{S^{\prime }}=R\cdot \vec{S}$$

However, inverting **R** is not trivial since it is quasi-singular, making the problem ill-posed. Moreover, the problem is further complicated by the presence of a noise term which modifies Equation (2) into

(3) $$\vec{S^{\prime }}=R\cdot \vec{S}+\vec{\varepsilon }$$

**Figure 1:**
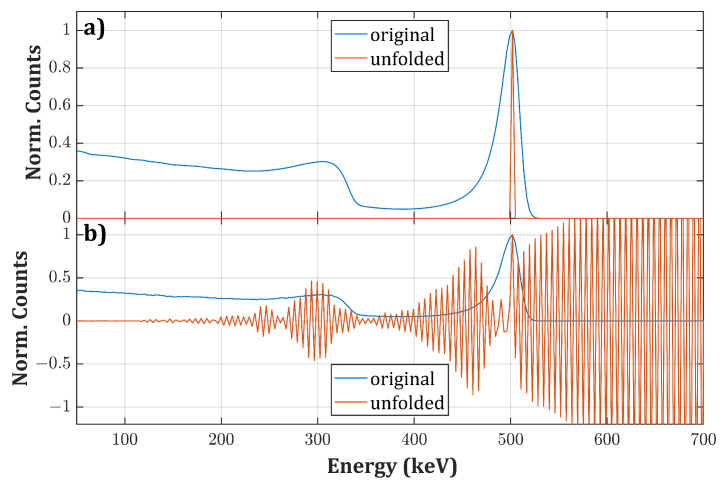
(**a**) Ideal synthetic spectrum (i.e., a column of **R**) for a monoenergetic radiation of 500 keV (blue curve) and the corresponding unfolded spectrum after applying **R**${}^{-1}$ (orange curve); (**b**) same spectrum after adding a slight Gaussian noise (blue curve) and the corresponding unfolded spectrum after applying **R**${}^{-1}$ (orange curve).

If vectors $\vec{S}$ satisfying $R\cdot \vec{S}=0$ or $R\cdot \vec{S}=\vec{\varepsilon }*$ exist, where $\vec{\varepsilon }*$ is negligible with respect to $\vec{\varepsilon }$, they can be added to the true physical solution and Equation (3) would still be satisfied. However, since the problem is unstable for small fluctuations, these additional terms may dominate with respect to the true spectrum $\vec{S}$, thus making the number of potential solutions infinite within error bounds [7]. As a consequence, if we just apply $R^{-1}$ to a measured spectrum, small uncertainties cause large oscillations and non-physical features in the unfolded spectrum (Figure 1).

The mathematical complexity of the problem encouraged the scientific community to develop innovative and robust methods to invert Equation (2) in order to find *S*. Different methods have appeared on the scene over the decades which can be gathered into various categories (direct inversion [9], least-squares [10,11], Monte Carlo [12,13], iterative [14,15,16,17,18,19], and Bayesian [20,21,22]). Recently, thanks to the increasing popularity of machine learning, alternative approaches have been proposed for unfolding neutron spectra (neural networks [23,24,25,26] and genetic algorithms [27,28]). This class of algorithms certainly allows for facing the problem in a completely new way. Here, we propose an approach based on the Genetic Algorithm (GA) which is inspired by the Darwinian theory of natural selection. This type of algorithm is a branch of the evolutionary computation family and is usually employed in the optimization and search problem. Here, the task of GA is neither inverting **R** nor finding the mathematical solution to this problem: it searches for an approximate and physically reasonable solution which best matches the measured spectrum. As a matter of fact, the metaheuristic procedure performed by a GA allows for speeding up the solution search with respect to a Monte Carlo approach. At the same time, it is characterized by a random component which permits a better exploration of the solution space with respect to other iterative and predetermined methods, where, given a certain input, the algorithm always converges to the same output [15,17,18]. The stochastic optimization performed by GAs is extremely fast and accurate even without prior assumptions. Moreover, we propose an approach to further accelerate the search by choosing a proper initialization in order to direct the algorithm on the right path. The algorithm has been validated on actual experimental spectra of different radionuclides acquired with different detectors based on CdZnTe (CZT).

## 2. Materials and Methods

In this section, we outline the basic principles of GAs and how these procedures have been implemented in this work. Furthermore, we describe the experimental conditions and the characteristics of the CZT detectors employed to acquire the γ-spectra.

### 2.1. Genetic Algorithm

GAs, as already briefly mentioned, emulate the process of selection performed by a hostile environment on a population of individuals. The ones with the best characteristics are more likely to pass down their gene pool to the offspring, whereas weak individuals die without reproducing (“survival of the fittest”). As a result, the overall fitness of the population will improve through generations. In computer science, the individuals (or solutions) are arrays of bits/integers/real numbers that undergo four basic operations:

- production the first generation of individuals (initialization)
- selection of individuals for mating (selection)
- generation of new individuals (crossover)
- modification of the genome (mutation)

**Figure 2:**
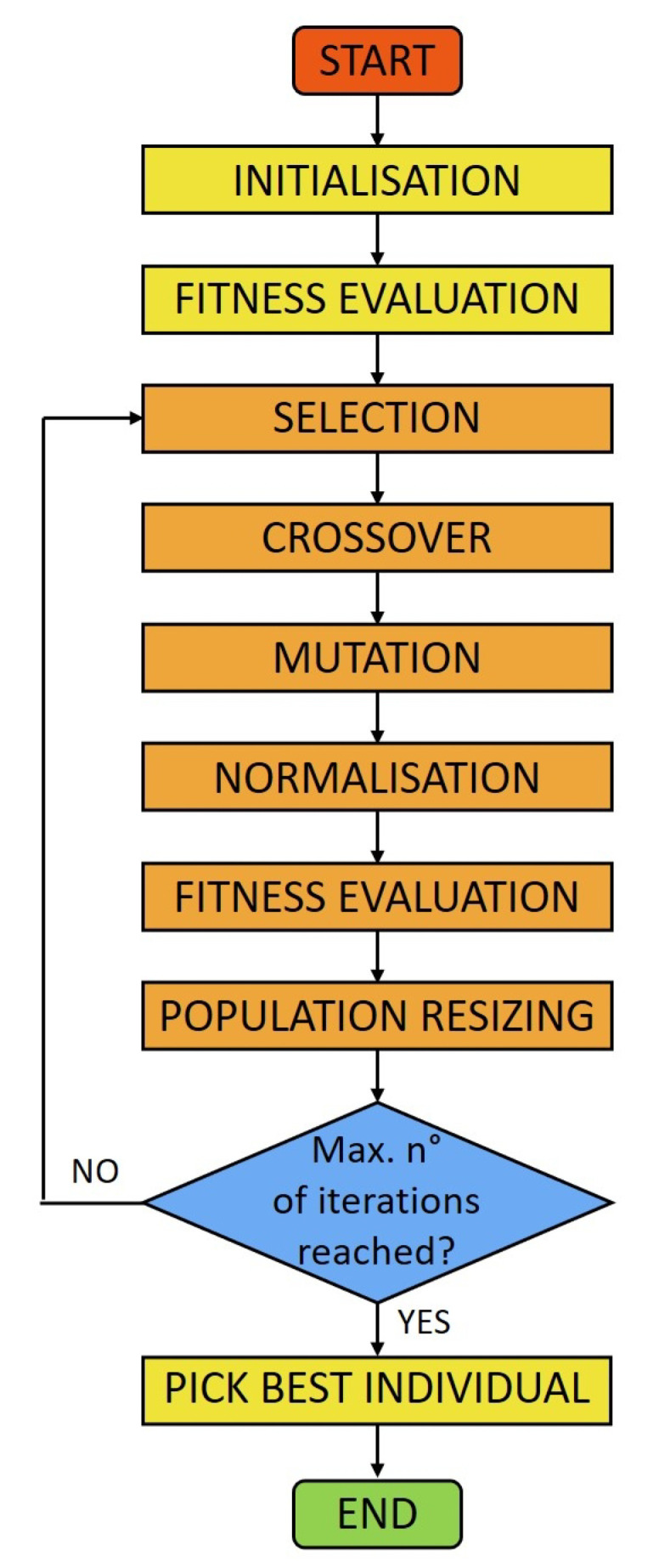
Flowchart of the GA used in this work.

In the present work, the individuals represent possible incident energy PDF. They consist of vectors of non-negative values with a unitary area whose length *L* is equal to the length of the measured spectrum. Their genes, which are the normalized number of counts in each channel, determine how the measured spectrum would appear through the convolution with the response function R. Thus, the fitness, which represents the objective function, is defined as

(4) $$\mathrm{fitness}=\mathrm{RSS}=\frac{1}{L}\sum_{i=1}^{L}{\left(\sum_{j=1}^{L}R_{ij}\cdot S_{j}^{*}-S_{i}^{\prime }\right)}^{2}$$

where $S^{*}$ is a potential solution. Therefore, the best individual is the one that minimizes the normalized residual sum of squares (RSS) between the observed spectrum $S^{\prime }$ and its convolution with **R**. In order to maximize the exploration of the search space, the initial population is randomly generated, and it is composed of $N_{s}$ individuals. The selection is performed by running a number of “tournaments” given by the $N_{s}\cdot P_{c}$ product, where $P_{c}$ is the crossover probability (see Table 1). The fitnesses of the competitors are compared, and the one with the lower *f* obtains the right of procreating:

(5) $$winner=arg\;min(f\left(\vec{S}{}^{*}\right),f\left(\vec{S}{}^{**}\right))$$

where $\vec{S}{}^{*}$ and $\vec{S}{}^{**}$ are two randomly selected individuals. Then, the $N_{s}\cdot P_{c}$ selected individuals are combined in pairs to produce an equal number of offspring: given two parents ${\vec{P}}_{1}$ and ${\vec{P}}_{2}$ (i.e., the winners of two different tournaments), the offspring ${\vec{O}}_{1}$ and ${\vec{O}}_{2}$ are defined as the weighted average of the parents:

(6) $$\begin{array}{c} {\vec{O}}_{1}=w{\vec{P}}_{1}+\left(1-w\right){\vec{P}}_{2}\\ {\vec{O}}_{2}=\left(1-w\right){\vec{P}}_{1}+w{\vec{P}}_{2} \end{array}$$

where 0<w<1 is a constant. Subsequently, a number of genes, given by the product $P_{m}\cdot L$ where $P_{m}$ is the mutation probability, is randomly selected in each new generated solution for the mutation: their values are replaced with random ones in the interval $\left[0,m\cdot v_{i}\right]$, where $v_{i}$ is the value in the selected *i*-th channel, and *m* is a tuned parameter (see Table 1). Usually, at this point, a smoothing step is required since highly oscillating solutions can still give good results because of the quasi-singularity of **R** [28], although this operation would also broaden true peaks. However, in this case, smoothing is not required thanks to the choice of the crossover method, which allows for attenuating great differences among individuals without smearing out the real characteristic lines. After normalizing the area of the mutated offspring, the fitness is again evaluated for all the new individuals and the entire population is resized by discarding the worst ones (elitist selection). The algorithm stops after a maximum number of iterations or when *f* falls below a certain threshold. As the last step, each bin is multiplied by a gain which accounts for the photons which pass through the crystal without interacting. This information is provided by the simulation tool used to obtain **R** (see the next section). The values of the parameters used in each step are reported in Table 1, and the flow chart of the code is shown in Figure 2. A calibration procedure has been performed to tune them to obtain the best overall performances in terms of running time (time required per each generation) and convergence speed (number of generations required to reach the goal). The algorithm has been implemented in MATLAB and has been tested on a standard single-core laptop.

### 2.2. Experimental Validation

We tested the algorithm on experimental spectra acquired with detectors with different size and contact geometry.

**Figure 3:**
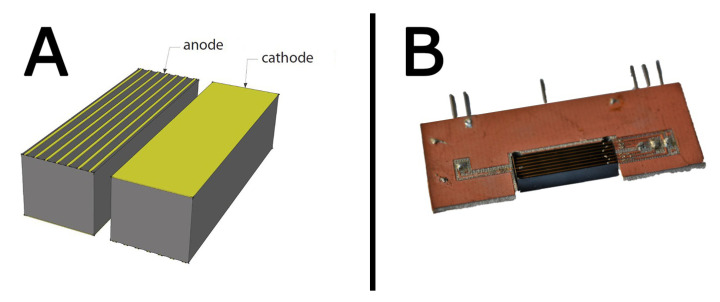
(**A**) 3D model of the drift strip detector; (**B**) actual CZT detector bonded to the intermediate electronic board.

The first detector consists of a 20×4.5×6 mm${}^{3}$ crystal realized by reprocessing standard spectroscopic grade CZT material purchased from Redlen Technologies (Canada). The crystal presents a strip electrode geometry: the anode is segmented in seven 250 μm strips with an intergap of 550 μm, whereas the cathode is full area (Figure 3). Contacts were realized with gold electroless deposition in aqueous solution. Starting from the most external to the central one, the strips were polarized at −450 V, −300 V, −150 V, and 0 V, respectively, and the cathode was biased at −450 V. This geometry allows for drifting the photogenerated carriers towards the collecting central strip and guarantees a high energy resolution above 100 keV regardless of the irradiation direction. The analog readout system was developed by due2Lab s.r.l. The readout channel includes a Cremat CR110 CSP (decay time =140 μs), a shaper amplifier (shaping time =2 μs), and a peak detector. The energy resolution is 3.9% FWHM @662 keV.

**Figure 4:**
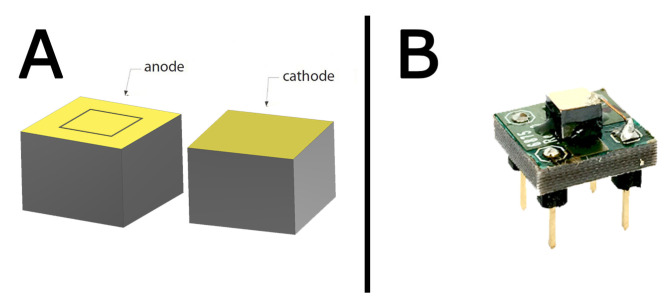
(**A**) 3D model of the single pixel detector; (**B**) actual CZT detector bonded to the intermediate electronic board.

The second detector consists of a 4.1×4.1×2.8 mm${}^{3}$ detector obtained by reprocessing a standard CZT crystal purchased from Redlen Technologies (Canada) with a full-area cathode, a 2×2 mm${}^{2}$ pixel, and a guard ring on the anode with a gap of 50 μm (Figure 4). Sensor refabrication has been carried out by due2lab s.r.l.; gold contacts were fabricated using the electroless deposition process from alcoholic solution as described in [29,30]. The detector was biased at −850 V, and it was irradiated from the cathode side. The CZT sensor unit (D2L001) and the single-channel digital pulse processor unit (D2L009-1) are part of the Hyperspectral X-ray Spectrometer (HXS) developed by due2lab. The energy resolution is 4.0% FWHM @60 keV.

The response function $R\left(E,E^{\prime }\right)$ of both detectors was obtained using the simulation tool described in [31], which considers each step of the signal generation process, from the radiation–matter interaction to the charge transport to the signal induction on contacts. We tested the algorithm on four different radioactive sources: ${}^{137}\mathrm{Cs}$ and ${}^{133}\mathrm{Ba}$ with the drift strip detector; ${}^{241}\mathrm{Am}$ and ${}^{57}\mathrm{Co}$ with the single pixel detector. Experimental spectra were re-binned on the energy vector of R (256 bins in the 0–150 keV and 0–750 keV energy range for the single pixel and drift strip detectors, respectively).

## 3. Results

In this section, we present the experimental validation of the proposed algorithm on actual γ-spectra of four different radionuclides: ${}^{137}\mathrm{Cs}$, ${}^{133}$Ba, ${}^{241}\mathrm{Am}$ and ${}^{57}\mathrm{Co}$. The best individual in the population after $10^{4}$ generations is taken as the final solution (referred to as unfolded spectrum) which is compared to the tabulated intensities for emission of *X*- and γ-photons in the case of unshielded radioactive sources [32]. In the case of ${}^{241}\mathrm{Am}$, the $X_{L}$-ray emissions’ intensities are compared to the ones reported in [33] (self-fluorescence of ${}^{241}\mathrm{Am}$ and scattered bumps are not considered). Tabulated intensities are normalized with respect to the main photopeak. The convolution of the solution with R (referred to as the folded spectrum) is instead compared to the measured spectrum. We observed that a number of iterations greater than $10^{4}$ would not improve the fitness because of non-idealities of **R** and the presence of experimental noise. Thus, the reported RSS values represent the lower achievable limit. The PhotoPeak Enhancement (PPE) is defined as the ratio between the intensities of the main photopeaks of each source before and after applying the algorithm. Results are summarized in Table 2.

**Figure 5:**
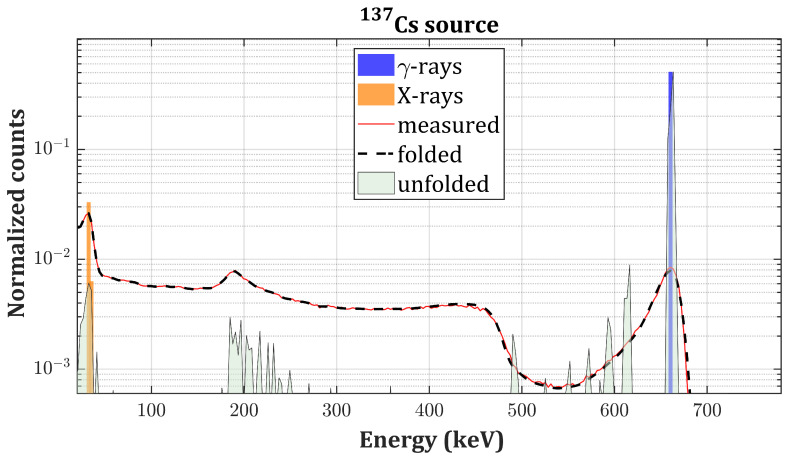
${}^{137}\mathrm{Cs}$ unfolded spectrum (gray curve) compared to the measured spectrum (red curve), the folded spectrum (black dashed curve), and the tabulated intensities of the γ-emissions (blue bars) and *X*-emissions (orange bars) of ${}^{137}\mathrm{Cs}$ on a logarithmic scale.

**Figure 6:**
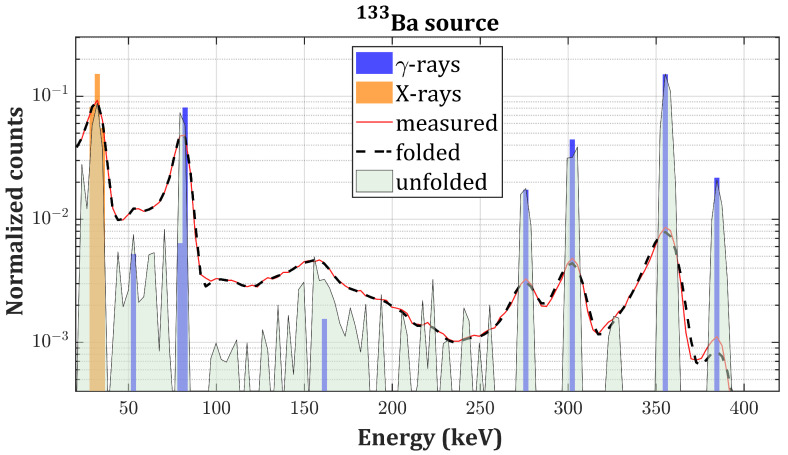
${}^{133}\mathrm{Ba}$ unfolded spectrum (gray curve) compared to the measured spectrum (red curve), the folded spectrum (black dashed curve) and the tabulated intensities of the γ-emissions (blue bars) and *X*-emissions (orange bars) of ${}^{133}\mathrm{Ba}$ on a logarithmic scale.

**Figure 7:**
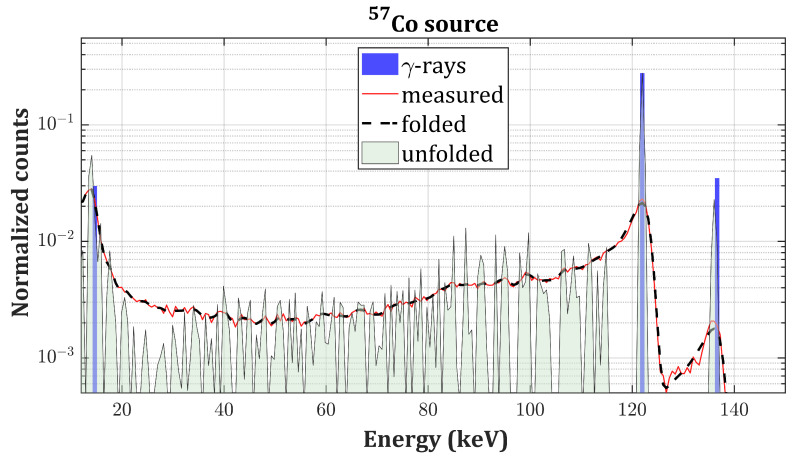
${}^{57}\mathrm{Co}$ unfolded spectrum (gray curve) compared to the measured spectrum (red curve), the folded spectrum (black dashed curve) and the tabulated intensities of the γ-emissions (blue bars) of ${}^{57}\mathrm{Co}$ on a logarithmic scale.

**Figure 8:**
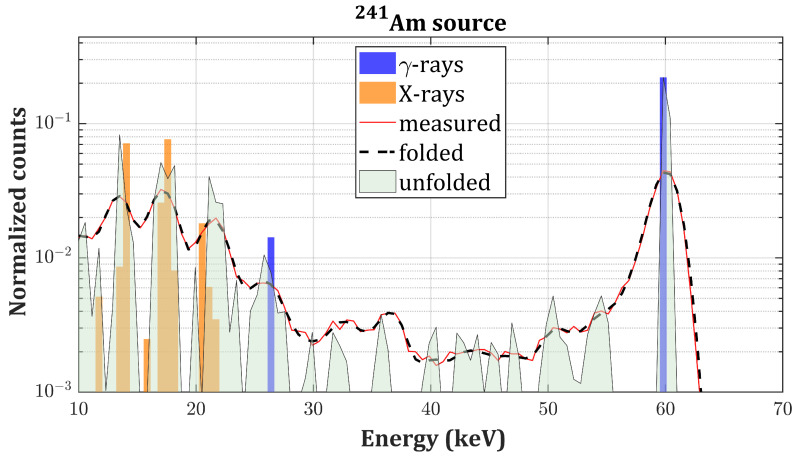
${}^{241}\mathrm{Am}$ unfolded spectrum (gray curve) compared to the measured spectrum (red curve), the folded spectrum (black dashed curve) and the tabulated intensities of the γ-emissions (blue bars) and *X*-emissions (orange bars) of ${}^{241}\mathrm{Am}$ on a logarithmic scale.

Figure 5 and Figure 6 present the results of the deconvolution of ${}^{137}\mathrm{Cs}$ and ${}^{133}\mathrm{Ba}$ γ-spectra, respectively, measured with the drift strip detector (Figure 3). Figure 7 and Figure 8 present the results of the deconvolution of ${}^{57}\mathrm{Co}$ and ${}^{241}\mathrm{Am}$ γ-spectra, respectively, measured with the single pixel detector (Figure 4).

## 4. Discussion

The unfolding process based on GA successfully removed escape peaks, Compton edges, and continua and peak broadening from the γ-spectra of four different radionuclides (${}^{57}\mathrm{Co}$, ${}^{241}\mathrm{Am}$${}^{133}\mathrm{Ba}$, and ${}^{137}\mathrm{Cs}$), and the unfolded spectra consists of δ-like peaks. PPE values indicate a remarkable improvement of the signal-to-noise-ratio. By increasing the energy, the extent of Compton scattering grows, hence the enhancement is more evident. First of all, it should be emphasized that these results prove and confirm the accuracy of the simulation tool used to produce the response function **R** [31]. Secondly, this approach effectively takes advantage of all the information contained in the measured spectra to reconstruct and enhance even the weakest photopeaks without prior assumptions about the incident radiation.

Regarding the drift strip detector, the relative intensities of the unfolded peaks are in good agreement with the tabulated ones except in the 0–30 keV energy region (probably because of a partial shielding from the detector case), and the folded spectra remarkably match the measurements. However, spiky residuals are still present on the low energy side of the photopeaks in the 500–600 keV and in the 200–250 keV energy ranges in Figure 5b and Figure 6b, respectively. This is due to the non-idealities of the response matrix, which partially fails to reproduce the effect of double Compton events, the only ones which could produce counts in these energy ranges. The simulator underestimates the extent of this effect, and additional peaks are required to match folded spectra with the measurements. Furthermore, the backscattering peaks are still present after the deconvolution in the 190–300 keV energy range in Figure 5b and in the 120–190 keV energy range in Figure 6b. The reason relies on the fact that the response matrix was obtained by simulating the interaction of the radiation with a bare CZT crystal. The absence of components in the physical surroundings of the detector (e.g., metallic case, electronic read out) prevented the formation of this feature in the simulated spectra. Thus, the algorithm interprets the backscattering peak as an actual photon source. The overall shape and intensity of the backscattering peaks are indeed preserved in the unfolded spectra (without the noise broadening) because it is not considered as an artifact introduced by the instrument. This could be easily fixed by simulating the interaction with the whole detector.

The results of the unfolding process applied to the spectra measured with the single pixel detector are slightly less satisfactory. As a matter of fact, the simulation tool does not consider the possibility of the photogenerated charge cloud to be collected by multiple electrodes (“charge sharing effect”). In this case, this phenomenon occurs when a photon interacts in the volume below the gap between the pixel and the guardring. After drifting towards the anode, the cloud is shared among these two contacts and the amount of charge collected by the pixel (i.e., what we actually measure) depends on the distance of the cloud from the center of the pixel. The extent of these losses spans from 0 to the full energy of the photon, hence producing residuals in the whole energy range up to the photopeak position: this is approximately what we observe in the unfolded spectra in Figure 7 and Figure 8. The algorithm struggles to reconstruct the intermediate region, and the final RSS values are slightly higher with respect to the drift strip detector.

**Figure 9:**
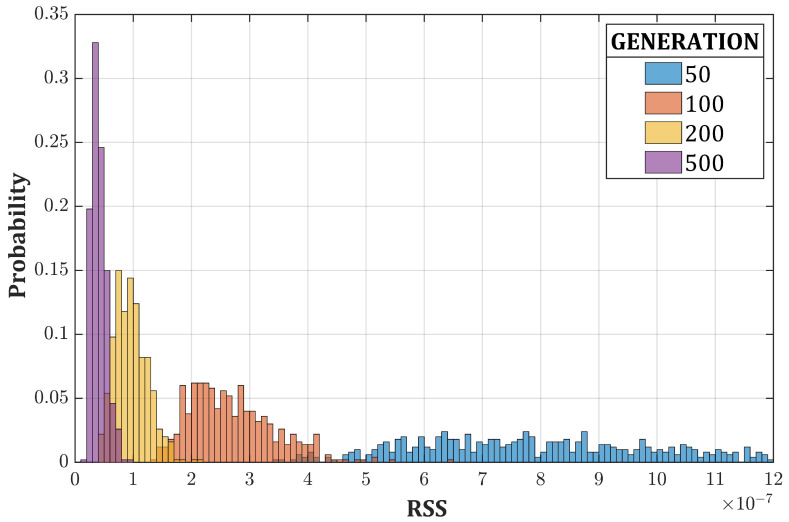
Probability density function of the RSS for the ${}^{137}\mathrm{Cs}$ spectrum as a function of the number of iterations for the standard GA (500 run each).

We demonstrated the robustness of the algorithm by obtaining remarkable results even starting from randomly generated candidate solutions. We stress the fact that the algorithm is able to reproducibly find the best approximate solution, provided that a sufficient number of generations has passed as shown in Figure 9, where the RSS of the ${}^{137}\mathrm{Cs}$ unfolded spectrum is reported as a function of the number of generations. This is indeed a peculiarity of GAs: if the random component (selection, crossover and mutation) is correctly calibrated, local minima do not represent an obstacle.

**Figure 10:**
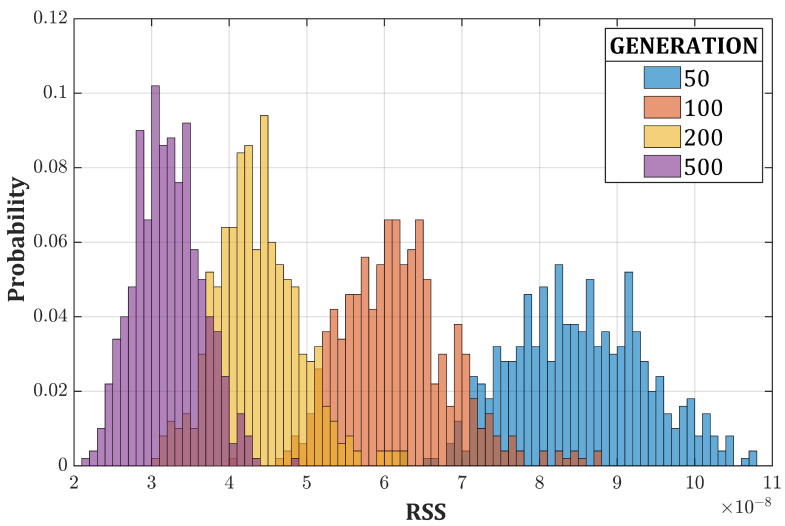
Probability density function of the RSS for the ${}^{137}\mathrm{Cs}$ spectrum as a function of the number of iterations for the boosted GA (500 run each).

However, at the beginning, the convergence speed is low. The reason relies on the fact that the GA starts from a completely random situation and the first few hundreds of iterations are required just to find good candidate solutions which are later refined. In the first generations, the crossover is less effective since randomly generated individuals are combined, thus obtaining nearly equivalent solutions. An actual improvement can only be achieved when the correct genes are mutated (i.e., when peaks starts appearing), but this means that the main leading operation is the mutation whose extent is, however, quite limited. Mutating a larger number of genes would indeed stimulate the generation of better candidate solutions, but this would be detrimental in the second part where an excessive mutation would deteriorate the new individuals. A possible solution consists of using a variable mutation parameter $P_{m}$ which decreases as the average fitness improves (Adaptive GA), but it would be difficult to finely tune $P_{m}$ and *m* in combination with $P_{c}$ to obtain the best overall performances. Instead, we propose to exploit the information contained in the measured spectra to overcome this problem by smartly initializing the population. This procedure is referred to as “seeding”, and it consists of inserting suitable guesses in the first generation to direct the algorithm on the right path and to greatly increase the convergence speed. As a matter of fact, the measured spectrum still preserves several features of the true incident radiation (photopeaks), and it would definitely represent a valid guess. A much better candidate can actually be obtained by applying the iterative method described in [15]:

(7) $$S^{n+1}=\frac{S^{\prime }}{R\cdot S^{n}}\cdot S^{n}\;\mathrm{with}\;S^{0}=S^{\prime }$$

where $S^{n}$ and $S^{n+1}$ are the partially unfolded spectra after *n* and n+1 iterations, respectively. Our choice of this algorithm derives from its simplicity and, thus, its speed. After few iterations (5 in this case), Compton continua are reduced and the peaks are emphasized. Inserting only one partially unfolded individual in the initial population (RSS ∼5 × 10${}^{-7}$ in the case of the ${}^{137}\mathrm{Cs}$ spectrum) is enough because the crossover (Equation (6)) and the elitist selection facilitate a fast diffusion of the strong genes among the whole population. In this way, the crossover is extremely effective since the beginning, and we achieved the goal of guiding the algorithm without excessively restricting the search process, which could lead to local optima (Figure 10). The performances (RSS average values and standard deviations) of Figure 9 and Figure 10 are summarized in Table 3.

## 5. Conclusions

An unfolding approach of γ-spectra based on GA is proposed. The experimental validation was performed by unfolding of the spectra of four radionuclides: ${}^{137}\mathrm{Cs}$ and ${}^{133}\mathrm{Ba}$ measured with the drift strip detector; ${}^{57}\mathrm{Co}$ and ${}^{241}\mathrm{Am}$ measured with the single pixel detector. The unfolded spectra successfully consist of δ-like lines in correspondence with the characteristic γ- and *X*-emissions of each source, and the relative intensities are in good agreement with the tabulated ones. The physical explanation of the residuals present after the deconvolution process is given. Fundamentally, the limits of this approach derive from the accuracy of the simulation toolkit used to calculate the spectral response matrix, provided that statistical noise is negligible in the energy region in which the relevant features are present. Studies are ongoing to consider the charge sharing effect in the simulation of **R**, hence allowing to extend the approach on pixelated detectors. The application of GA in this context allows for bypassing the problem of inverting **R**, hence avoiding the need of regularizations. The simplicity of the operations used in this work ensures low computation and high speed, thus allowing its implementation on a Field Programmable Gate Array. Furthermore, a method to boost the search process is proposed to effectively exploit the physical information contained in the measured spectra.

This approach can be extended to different classes of radiation detectors where it is possible to accurately simulate the response function of the device.

## Figures and Tables

**Table 1:** Description and values of the parameters used in the GA.

Parameter	Description	Value
$N_{s}$	Size of the population	50
$P_{c}$	Crossover probability	0.8
$P_{m}$	Mutation probability	0.01
*w*	Crossover weight	0.3
*m*	Mutation upper bound	3

**Table 2:** RSS and PPE values for each deconvoluted spectra reported in this work.

Isotope	Detector	RSS	PPE
${}^{137}\mathrm{Cs}$	Drift strip	$1.5\times 10^{-8}$	64.8
${}^{133}\mathrm{Ba}$	Drift strip	$7.0\times 10^{-8}$	17.6
${}^{57}\mathrm{Co}$	Single pixel	$7.8\times 10^{-8}$	12.0
${}^{241}\mathrm{Am}$	Single pixel	$9.8\times 10^{-8}$	4.9

**Table 3:** Average value and standard deviation of RSS and average running time as a function of the number of iterations (500 run each) for both standard and seeding GA.

Standard GA			Seeding GA		
**N${}^{\circ }$ of** **Generations**	**RSS $\times 10^{8}$**	**Av. Running** **Time (ms)**	**N${}^{\circ }$ of** **Generations**	**RSS$\times 10^{8}$**	**Av. Running** **Time (ms)**
50	83±25	84	50	8.6±1.0	85
100	27±7	157	100	6.2±0.7	158
200	9.7±2.8	313	200	4.4±0.5	316
500	4.1±1.3	780	500	3.2±0.4	785

## Abbreviations

The following abbreviations are used in this manuscript:

RTSD	Room Temperature Semiconductor Detector
GA	Genetic Algorithm
CZT	Cadmium Zinc Telluride
RSS	Residual Sum of Squares
CSP	Charge Sensing Preamplifier
PPE	PhotoPeak Enhancement
